# Effect of Caudal Keel Structure on the Head Stability of a Bionic Dolphin Robot

**DOI:** 10.3390/biomimetics10110756

**Published:** 2025-11-10

**Authors:** Weijie Gong, Yanxiong Wei, Hong Chen

**Affiliations:** College of Mechatronics and Control Engineering, Shenzhen University, Shenzhen 518060, China; 2310294005@email.szu.edu.cn (Y.W.); chenhong@szu.edu.cn (H.C.)

**Keywords:** caudal keel, bionic dolphin robot, head stability, self-propulsion, CFD

## Abstract

To address the challenge of head stability in a biomimetic robotic dolphin during self-propulsion, this study systematically investigates the passive stabilization mechanism of a bio-inspired caudal keel. A combined experimental and computational fluid dynamics (CFD) approach was employed to evaluate four keel geometries across a tail oscillation frequency range of 0.5–2 Hz. The experimental results demonstrate that the optimal keel configuration reduced the standard deviation of the head pitch angle by 20.9% at 2 Hz. CFD analysis revealed a dual stabilization mechanism: an effective keel not only attenuates the intensity of the primary disturbance moment at the driving frequency but, more critically, also enhances the spectral purity of the signal by suppressing high-frequency harmonics and broadband stochastic noise through the systematic reorganization of caudal vortices. A systematic investigation of keel geometry identified non-dimensional height (h/c) as the dominant parameter, with its stabilizing effect exhibiting diminishing returns beyond an optimal range. Furthermore, a quantifiable design trade-off was established, showing an approximate 9.1% increase in the Cost of Transport (CoT) for the most stable configuration. These findings provide quantitative design principles and a deeper physical insight into the passive stabilization of biomimetic underwater vehicles, highlighting the importance of both disturbance intensity and spectral quality.

## 1. Introduction

In cutting-edge domains such as marine exploration and underwater operations, biomimetic autonomous underwater vehicles (AUVs) have become a focal point of research due to their potential advantages in maneuverability, efficiency, and stealth. By mimicking the highly efficient locomotion of marine life, these bio-inspired vehicles surpass the limitations of energy consumption and acoustic noise associated with traditional propeller-based systems [1,2]. However, despite their theoretical promise, a significant challenge impedes the translation of biological principles into reliable engineering applications: attitude instability during motion [3,4].

Specifically, during rectilinear swimming, the periodic oscillation of the tail fin generates complex unsteady hydrodynamic forces. These forces inevitably induce responsive moments at the vehicle’s anterior, leading to undesirable pitching and yawing oscillations of the head [5,6,7]. This “head oscillation problem” not only increases fluid drag and reduces propulsive efficiency but also degrades the performance of high-precision onboard sensors such as cameras and sonar [8]. Enhancing a robot’s stability directly improves its operational capabilities, as a stable platform allows sensors to be closer to targets without the risk of accidental crashes, thereby significantly increasing the reliability of autonomous inspection, intervention, and navigation tasks [9]. Among these instability modes, pitching oscillations induced by vertical moments are particularly pronounced, as they directly impact the forward-looking sensing attitude and overall energy efficiency, representing a critical challenge in contemporary biomimetic AUV design [10,11,12].

**Figure 1:**
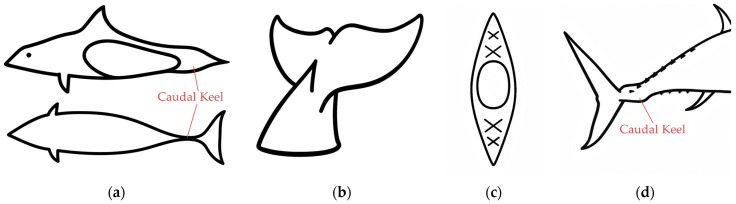
Caudal keel structure of high-speed swimmers. (**a**,**b**) Caudal keel of Phocoenoides dalli; (**c**) cross-section of the caudal peduncle of Harbor Porpoise; (**d**) caudal keel of yellowfin tuna.

High-speed marine animals offer valuable insights into resolving this stability issue. For instance, the caudal keel, found in species like the Dall’s porpoise, is considered a key passive stabilization structure, as illustrated in Figure 1 [13,14,15]. Although the potential of the caudal keel for optimizing flow fields and enhancing stability is widely recognized [16], existing research has predominantly focused on macro-level analyses of its propulsive performance or the application of active control strategies [8,17]. Effective, active control methods have inherent drawbacks, including high-energy consumption, system complexity, and reliance on sensors, which limit their viability for long-endurance missions. In contrast, passive stabilization methods derived from biological morphology exhibit considerable potential, owing to their zero energy consumption, high reliability, and structural simplicity [18,19].

Despite this potential, the hydrodynamic mechanisms by which the caudal keel effectively suppresses head disturbances remain underexplored. This paper aims to fill this knowledge gap by investigating the influence of the caudal keel on the head stability of a biomimetic robotic dolphin across various frequencies. Through a combination of experimental analysis and CFD simulations, we seek to elucidate its core stabilization mechanism. This systematic investigation aims to provide a theoretical foundation and quantitative design criteria for the next generation of biomimetic AUVs engineered for both high stability and superior maneuverability.

## 2. Experimental and Numerical Methods

### 2.1. Experimental Investigation

#### 2.1.1. Robotic Platform and Experimental Setup

**Figure 2:**
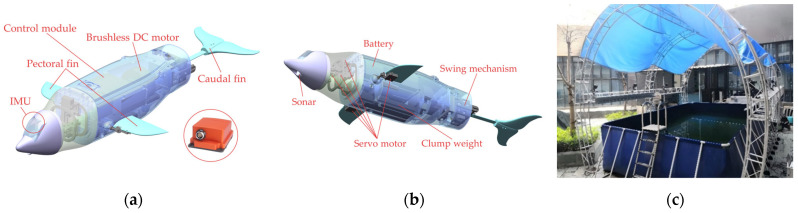
The bionic dolphin robot prototype and the experimental platform: (**a**) The CAD model of robotic dolphin with IMU; (**b**) The CAD model of robotic dolphin; (**c**) Water tank.

The experimental platform used in this study is an in-house-developed biomimetic robotic dolphin, with its mechanical design illustrated in Figure 2a,b. Inspired by a real dolphin, the robot features a streamlined body to reduce hydrodynamic drag [17]. Its modular fuselage is primarily composed of a head, a pair of two-joint pectoral fins, a single-joint caudal fin, and a rigid main torso [15]. For this research, the motor-driven, single-joint, lunate-shaped caudal fin served as the sole source of both propulsion and hydrodynamic disturbance.

As propulsion originates from the vertical oscillation of the caudal fin, this study focuses on the type of instability that it primarily induces, namely the pitching motion. To precisely isolate and evaluate the passive stabilization effect of the caudal keel, all other actively controllable components were locked prior to the experiments. Specifically, the output shafts of the servo motors located in the head and pectoral fins—used for pitch and yaw control—were fixed to ensure a rigid connection with the main torso throughout all trials. This experimental setup guarantees that any observed fluctuations in head attitude are solely attributable to the variations in hydrodynamic moments generated by the periodic oscillation of the caudal fin.

For accurate measurement of head attitude, an Xsens MTi-100 Inertial Measurement Unit (IMU), with a sampling frequency of up to 400 Hz, was installed inside the head module. All swimming experiments were conducted in an indoor water tank with dimensions of 6.0 m × 4.0 m × 1.5 m (Figure 2c). The quiescent tank environment provided stable hydrodynamic conditions for testing. During the experiments, we systematically compared the motion characteristics of the four caudal keel configurations (No Keel, Normal Keel, Half-Height Keel, and Half-Width Keel) at four oscillation frequencies (0.5 Hz, 1 Hz, 1.5 Hz, and 2 Hz). To ensure the reliability and repeatability of the experimental results, a total of *n* = 5 independent trials were conducted for each experimental condition.

#### 2.1.2. Caudal Keel Design and Fabrication

**Figure 3:**
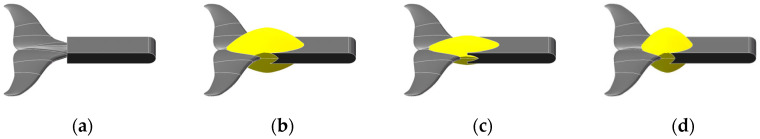
Geometric designs of the four caudal keel configurations: (**a**) No Keel; (**b**) Normal Keel; (**c**) Half-Height Keel; and (**d**) Half-Width Keel.

To systematically investigate the influence of the caudal keel’s geometry on stability, this study involved the design and testing of four distinct configurations: a baseline model without a keel (Figure 3a) and three biomimetic keels with varying geometric parameters (Figure 3b–d). The design of these biomimetic keels was inspired by anatomical observations of Dall’s porpoise caudal keel [11].

In the design process, the keel’s cross-section and longitudinal profile were simplified to an elliptical shape [20]. This approach not only preserves the core streamlined features of the biological prototype but also facilitates subsequent parametric control and precise fabrication. Using the standard Normal Keel as a baseline, we varied two key dimensionless parameters—the dimensionless height (h/c) and dimensionless width (w/c)—to evaluate their respective contributions to stability. These design configurations are illustrated in Figure 3, and their detailed geometric parameters are summarized in Table 1.

### 2.2. Numerical Simulation

#### 2.2.1. Geometric Model

**Figure 4:**
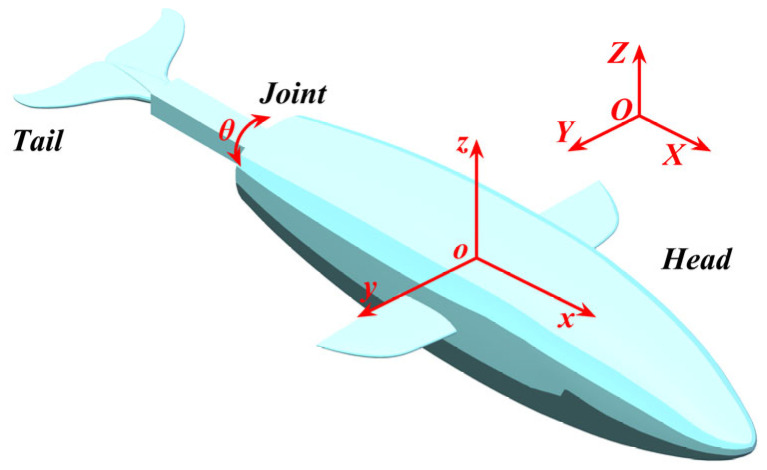
Geometric model of the bionic dolphin robot.

The geometry and dynamic behaviors of the biomimetic robotic dolphin in this study are represented by the 3D numerical model shown in Figure 4. The model is physically abstracted as a two-body system, comprising a rigid head and a rigid tail connected by a single-degree-of-freedom (1-DOF) rotational joint [21]. This rigid-body abstraction is a simplification of a real dolphin’s continuously flexible body and was chosen to isolate the specific hydrodynamic effects of the caudal keel. With a total length of 1010 mm, its external shape is designed to mimic the streamlined morphology of a real dolphin, ensuring that its hydrodynamic characteristics are biomimetically relevant.

To analyze the robot’s motion, two coordinate frames were defined: a fixed-body frame (o−xyz) attached to the dolphin’s overall center of mass (COM) and a stationary global frame (O−XYZ). Within the fixed-body frame, the origin, o, is located at the COM. The *x*-axis is aligned with the longitudinal axis of the body and is positive from tail to head; the *y*-axis is positive towards the starboard side; and the *z*-axis is directed vertically downward, forming a right-handed coordinate system. The head pitching motion, which is the focus of this study, is defined as the rotation about the *y*-axis.

#### 2.2.2. Computational Domain and Boundary Conditions

**Figure 5:**
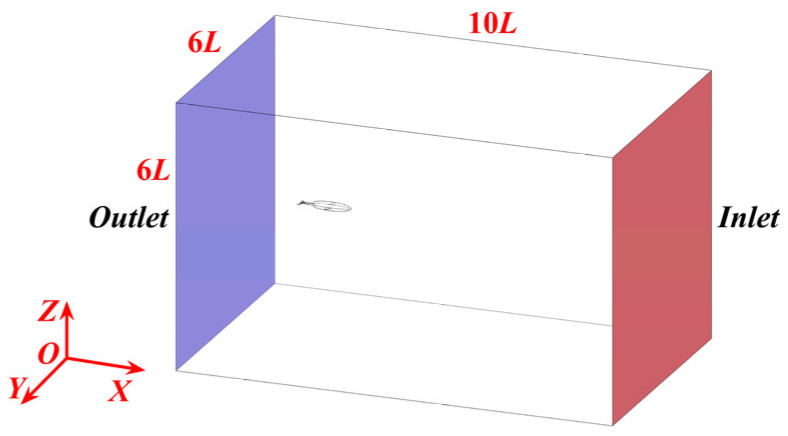
Computational domain.

The simulations were conducted in a virtual computational domain with dimensions of 10 L × 6 L × 6 L (where L is the robot’s length of 1.01 m) to minimize blockage effects. The domain inlet was defined as a velocity inlet; the outlet was prepared as a pressure outlet; and the surrounding far-field walls were set as symmetrical planes to approximate an open-water environment (Figure 5). This differed from the physical experiments, which were conducted in a confined 6.0 m × 4.0 m × 1.5 m water tank where wall effects, though minimized by the tank’s size relative to the robot, were inherently present.

#### 2.2.3. Governing Equations and Coupling Method

The numerical model is based on the geometry of the biomimetic robotic dolphin, as introduced in Section 2.1. During this study, the entire vehicle was divided into two rigid bodies: ① the head–torso section (hereafter referred to as the head), with its boundary surface denoted as *S*_H_; and ② the caudal peduncle-fin section (referred to as the tail), with its boundary surface denoted as *S*_T_. The head section only underwent pitching rotation about the *y*-axis of the overall center of mass (CG), and the tail section performed a relative oscillation with an angle of *θ*(*t*) within the fixed-body frame. The motion of the tail fin was affected by three components: a traveling wave motion relative to the body’s posterior end, the overall forward propulsion of the vehicle, and an oscillatory rotation about the *y*-axis. The equation for the dolphin’s body centerline is presented as follows [22,23]:

(1) $$z(k_{i},t)=A(\frac{0.21-0.66k_{i}}{1.1k_{i}^{2}+0.35k_{i}^{8}})\sin(2\pi ft)$$

where *z*(*k*_i_, t) is the amplitude function of points along the body axis as a function of time; *k*_i_ represents the ratio of the abscissa of each point on the body axis compared to the body length, *k*_i_ = *x*_i_*/L*_B_*, i =* 1, 2, 3,…; *f* is the oscillation frequency; and *A* is the oscillation amplitude of the tail fin. From Equation (1), the motion equation for the robotic dolphin’s tail fin is as follows:

(2) $$\theta (t)=\theta_{0}\sin(2\pi ft)$$

Three-dimensional incompressible continuity and Navier–Stokes (N-S) equations were primarily used to calculate fluid motion [24]:

(3) $$\begin{array}{c} \nabla \cdot u=0\\ \frac{\partial u}{\partial t}+(u\cdot \nabla )u=-\frac{1}{\rho }\nabla p+\frac{\mu }{\rho }\nabla^{2}u \end{array}$$

where ***u*** is the fluid velocity; *μ* is the dynamic viscosity of the fluid; *p* is the pressure; and *ρ* is the fluid density.

We employed the SST k-ω turbulence model, which is known for its robustness in handling complex flow separation problems [25]. For numerical discretization, a first-order implicit scheme was employed for the temporal term; second-order upwind and central difference schemes were applied to the convection and diffusion terms, respectively. The governing equations were solved iteratively within each time step using a pressure-based-coupled algorithm.

The motion of the robotic dolphin is defined as three-degree-of-freedom (3-DOF) swimming in the X-Y plane (comprising surge, sway, and pitch). Its dynamics are governed by the following equations of motion (EOMs):

(4) $$\begin{array}{c} m_{b}\dot{u}=F_{HX}+F_{TX}\\ m_{b}\dot{w}=F_{HZ}+F_{TZ}\\ I_{y}\dot{q}=M_{Y}=M_{HY}+M_{TY} \end{array}$$

The terms on the right-hand side (*F_HX_*, *F_TX_*, etc.) are the total hydrodynamic forces and moments exerted on the head (*S_H_*) and tail (*S_T_*) sections. These are obtained by integrating the local forces over the vehicle’s wetted surfaces. For any surface element *dS* ⊂ *S_H_ ∪ S_T_*, the differential hydrodynamic force *dF* resulting from the static pressure *p* and the viscous stress tensor τ is given by the following:

(5) dF=(−pn+τn)dS

The total forces and moments are then calculated by integrating the components of *dF* over the respective surfaces. The calculations for the head section are as follows:

(6) $$\begin{array}{c} F_{HX}=\iint_{S_{H}}dF_{X}\\ F_{HZ}=\iint_{S_{H}}dF_{Z}\\ M_{HY}=\iint_{S_{H}}\left(xdF_{Z}-zdF_{X}\right) \end{array}$$

The forces and moment on the tail section (*F_TX_*, *F_TZ_*, *M_TY_*) are calculated analogously by integrating over the surface *S_T_*.

In the EOMs, *m_b_* and *I_y_* are the mass and moment of inertia of the robotic dolphin about its overall center of mass (COM), respectively;$\dot{\;u}$ and $\dot{w}$ are translational accelerations in the *X* and *Z* directions; and $\dot{q}\;$is the pitching angular acceleration.

### 2.3. Numerical Verification

To ensure the accuracy of the numerical simulations, grid and time-step independence studies were conducted for the representative 2 Hz No Keel case.

The grid independence study is summarized in Table 2. When the grid was refined from medium (2.20 million cells) to fine (4.49 million cells), the change in the primary metric and pitching moment amplitude (*M*_Y_) was only 0.048%. Since this difference is negligible (well below 1%), the results are considered grid-independent. Therefore, to balance computational accuracy and cost, the medium grid was used for all subsequent CFD simulations.

Based on the selected medium grid, a time-step independence study was then performed (Table 3). Refining the time step from T/100 to T/200 resulted in a variation in only 0.024% for the pitching moment amplitude. This indicates that a time step of T/100 is sufficient to accurately capture unsteady flow characteristics. Consequently, a time step of T/100 was adopted for all simulations in this study.

### 2.4. Performance Metrics and Data Analysis

A set of quantitative metrics was employed to evaluate the robot’s stability and hydrodynamic performance.

To quantitatively assess head attitude stability, we adopted the standard deviation (*σ*) of the pitch angle as the primary metric. The smaller the value of this metric, which directly reflects the degree of dispersion of the pitch angle from the mean, the smaller the fluctuations in attitude and the higher the stability:

(7) $$\sigma =\sqrt{\frac{1}{N}\sum_{i=1}^{N}{\left(\theta_{i}-\bar{\theta }\right)}^{2}}$$

where *θ_i_* is the instantaneous pitch angle measured by the IMU at each sampling point; *θ* is the mean pitch angle for a given operational condition; and *N* is the total number of samples.

The hydrodynamic performance was analyzed using the non-dimensional Strouhal number (*St*) and Reynolds number (*Re*). These were based on characteristics such as the robot’s total body length (*L*) and its average forward velocity (*U*). The numbers are defined as *St = 2A_m_f/U* and *Re = ρUL/μ*, where 2*A_m_* is the transverse amplitude of the caudal fin [26].

## 3. Analysis of Experimental and Numerical Results

### 3.1. Head Stability Analysis: Experiment and Simulation

**Figure 6:**
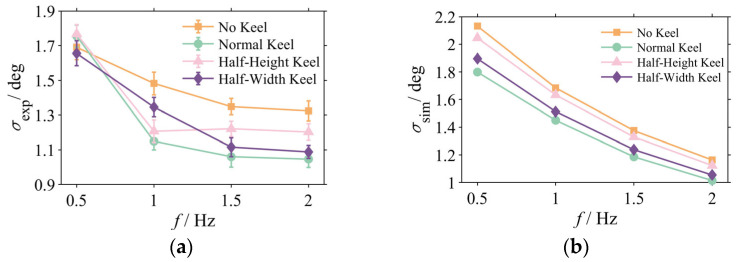
Standard deviation of head pitch angle versus frequency: (**a**) experimental results; (**b**) simulation results.

Figure 6 summarizes the mean standard deviation of the pitch angle for all experimental conditions, with error bars representing the standard deviation across *n* = 5 trials. The data clearly reveal that the influence of the caudal keel on stability exhibits a significant dependence on frequency. At the low frequency of 0.5 Hz, the differences in stability among the configurations are negligible, indicating that the keel provides no discernible advantage under this condition. However, as the oscillation frequency increases to 1 Hz and above, the stabilizing effect of the keel becomes apparent. In the high-frequency range of 1.5 Hz and 2 Hz, the Normal Keel and Half-Width Keel configurations demonstrate optimal stabilization performance.

Taking the 2 Hz case as a representative example, a detailed statistical analysis was performed. The Normal Keel configuration (M = 1.047°, SD = 0.048°) exhibited a significantly lower mean head pitch standard deviation compared to the No Keel configuration (M = 1.324°; SD = 0.058°). This represents an average stability enhancement of approximately 20.9%, and the difference was found to be highly statistically significant (t(8) = 8.24, *p* < 0.01), providing a strong experimental validation of its effectiveness.

To validate the fidelity of the numerical model and investigate the underlying mechanisms, the standard deviation of the head pitch angle was also calculated from the simulation results (Figure 6b). A comparison revealed good qualitative agreement between the simulation data (Figure 6b) and the experimental measurements (Figure 6a). Both datasets showed that the stabilizing effect of the keel is more pronounced at higher frequencies, and both correctly identified the Normal Keel as the most effective configuration. This strong correspondence validates the reliability of the established fluid–structure interaction (FSI) numerical model.

It is worth noting that the results of the physical experiments are subject to several sources of uncertainty. The high-performance specifications of the Xsens MTi-100 IMU employed (gyroscope noise density: 0.01 deg/s/√Hz) result in a substantially finer sensor precision than the measured improvements in stability. Other uncertainties include minor fabrication asymmetries and faint residual currents in the water tank. While these factors introduce variability, as reflected by the error bars in Figure 6a, the statistical analysis confirms that the observed performance differences, particularly at high frequencies, are significant and not attributable to random experimental noise. At these higher frequencies, the substantial gain in stability conferred by the keel far outweighs the influence of these uncertainties.

### 3.2. Attenuation and Decomposition of the Pitching Moment

With the numerical models being reliably validated, we can now examine the direct physical drivers behind the stability enhancement observed in the experiments. This section will first analyze the net pitching moment that drives the body’s oscillations and then decompose it to identify the ultimate stabilization mechanism.

#### 3.2.1. Analysis of Pitching Moment Amplitudes

**Figure 7:**
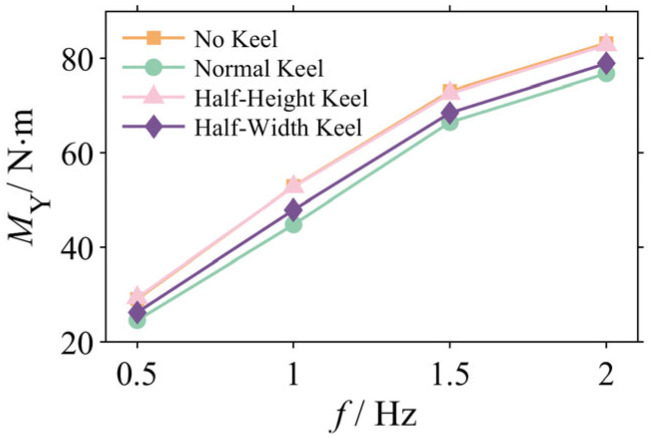
Simulation results of the amplitude of the pitching moment.

The root cause of the robotic dolphin’s pitching oscillations is the net pitching moment, *M_Y_*, acting on its body. Figure 6 illustrates the variation in the net pitching moment’s amplitude at various frequencies for the four keel configurations. A comparison between Figure 6 and Figure 7 reveals a strong negative correlation between the net moment amplitude and head attitude stability: the more stable the configuration, the smaller the net pitching moment experienced.

This correspondence is particularly evident in the mid-to-high frequency range (≥1 Hz). For instance, at 2 Hz, the Normal Keel configuration reduces the net pitching moment from 83.23 N·m (for the No Keel model) to 76.78 N·m, at an attenuation of 7.8%. This reduction in moment corresponds directly to the significant enhancement in head stability.

**Figure 8:**
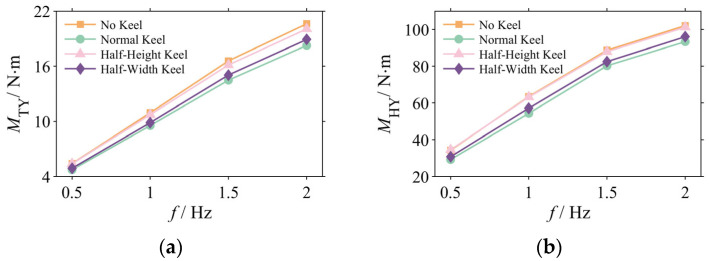
Amplitudes of the (**a**) disturbance moment from the tail (*M_TY_*) and (**b**) responsive moment from the head (*M_HY_*).

To pinpoint the source of the net moment attenuation, the moment was decomposed into two primary components: the disturbance moment from the tail (*M_TY_*) and the responsive moment from the head (*M_HY_*). Figure 8 illustrates the amplitude variations in these components.

The data reveal that the caudal keel attenuates both moment components; the effectiveness of this optimization aligns with the stability ranking that was previously established. At 2 Hz, the tail moment *M_TY_* (Figure 8a), which acts as the primary disturbance source, was reduced by the Normal Keel from 20.62 N·m to 18.25 N·m (an attenuation of 11.5%). The Half-Width and Half-Height keels provided attenuations of 8.3% and 2.7%, respectively. Correspondingly, the head moment *M_HY_* (Figure 8b), which is the hydrodynamic response, was also attenuated, and the Normal Keel reduced it from 102.11 N·m to 93.34 N·m (a reduction of 8.6%). This synergistic attenuation of both components ultimately reduces the net moment.

#### 3.2.2. Frequency Domain Analysis

The analysis reveals a disproportionate relationship, wherein a 7.8% attenuation in the net pitching moment amplitude at 2 Hz yielded a 20.9% improvement in head stability. This non-proportionality suggests that the stabilization mechanism may be closely linked not only to the overall reduction in disturbance amplitude but also to the spectral composition of the signal [27].

**Figure 9:**
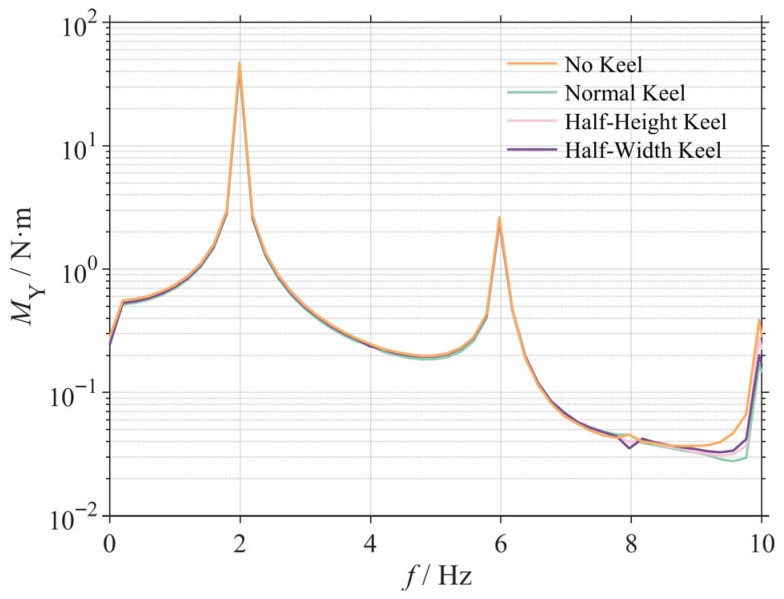
Frequency spectrum of the net pitching moment (*M_Y_*) at 2 Hz.

For the representative 2 Hz case (Figure 9), the spectral data indicate that the Normal Keel configuration enacts superior suppression across multiple frequency bands. Quantitative analysis confirms that its amplitude at a primary frequency of 2 Hz is 7.8% lower than that of the No Keel configuration. More significantly, the Normal Keel concurrently suppresses higher-frequency components, reducing the amplitude of the 6 Hz harmonic by 11.2% and the broadband noise floor in the 7–10 Hz range by 29%.

These findings substantiate a dual stabilization mechanism. An effective caudal keel not only attenuates the intensity of the primary periodic disturbance but also enhances the spectral purity of the moment signal by filtering out high-frequency harmonics and stochastic noise. This effective suppression of high-frequency components is considered a primary contributor to the observed disproportionate gain in head stability [28].

### 3.3. Mechanism Analysis: From Flow Field Reconstruction to Pressure Field Optimization

#### 3.3.1. Vorticity Field Analysis

**Figure 10:**
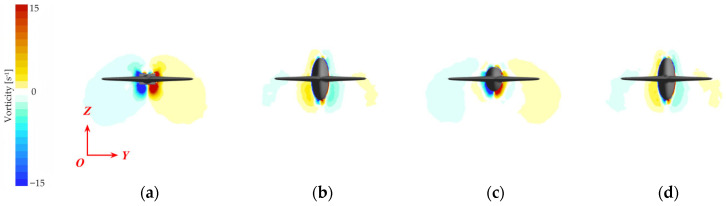
Cross-sectional streamwise vorticity contours of the caudal keel at 2 Hz: (**a**) No Keel; (**b**) Normal Keel; (**c**) Half-Height Keel; and (**d**) Half-Width Keel.

The core hydrodynamic function of the caudal keel is to fundamentally reconstruct the morphology of the vortex system at the tail, replacing chaotic and destabilizing vortex shedding with a pair of stable, symmetric, attached streamwise vortices [29]. This fundamental transformation, illustrated in Figure 10, serves as the physical origin of the keel’s stabilizing effect.

For the baseline No Keel model (Figure 10a), the rectangular cross-section with its sharp edges inevitably induces large-scale, unsteady flow separation during transverse motion. The resulting wake is filled with strong, randomly located vortex structures that lack axial coherence. This unpredictable vortex shedding is the direct cause of intense pressure fluctuations and the unsteady disturbance moment.

In contrast, all configurations equipped with a keel demonstrated varying degrees of flow optimization. Acting as a critical geometric constraint, the keel suppresses separation at the corners, allowing the shear layers to develop along its surface. Thus, the flow is reorganized into a more ordered vortex system. However, the effectiveness of this flow control differs significantly among the various keel geometries, a finding that aligns with the final stability ranking (Normal > Half-Width > Half-Height > No Keel).

A deeper analysis reveals that the contributions of the keel’s height and width to flow control are not equal. The height parameter is the key geometric element for achieving flow attachment, as it directly governs the keel’s ability to constrain transverse flow separation. The Half-Height Keel (Figure 10c), the least effective configuration, has a height of only 22 mm, which is insufficient to span the main separation region of the caudal peduncle. This leads to secondary separation at the keel’s upper edge, creating an additional source of vortex shedding, with large-scale shed vortices still present on both sides. In comparison, while the Half-Width Keel’s width is reduced to 25 mm, it retains a full height of 44 mm (Figure 10d). This height is sufficient to constrain the shear layers along its surface, effectively maintaining the basic state of flow attachment and promoting the formation of a more compact and symmetric vortex pair. The differentiating impact of geometric parameters explains why the moment attenuation of the Half-Width Keel (5.2%) is markedly superior to that of the Half-Height Keel (0.5%).

Ultimately, the optimal Normal Keel (Figure 10b), benefiting from the synergistic effect of its sufficient height (44 mm) and width (50 mm), successfully and completely reconstructs the chaotic vortex shedding into a pair of stable, coherent, highly symmetric, and attached streamwise vortices of lesser intensity, thereby sufficiently suppressing unsteady effects.

#### 3.3.2. Pressure Field Analysis and Its Coupling with Vorticity

**Figure 11:**
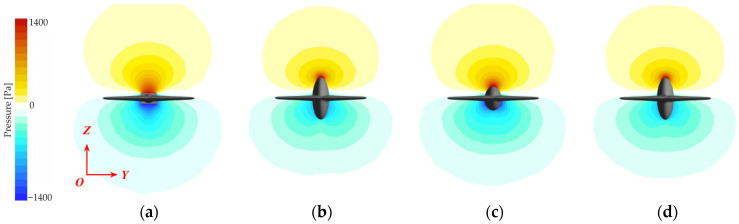
Pressure contours on the cross-section of the caudal keel at 2 Hz: (**a**) No Keel; (**b**) Normal Keel; (**c**) Half-Height Keel; and (**d**) Half-Width Keel.

The ordered vortex system reconstructed in the previous section has the direct physical effect of stabilizing the cross-sectional pressure field, transforming the originally pulsating and concentrated pressure distribution into a stable and smooth morphology. As illustrated in Figure 11, the optimization of the pressure field is the critical link connecting the field flow’s reconstruction to the ultimate moment attenuation.

For the baseline No Keel model (Figure 11a), the irregular vortex shedding process induces strong adverse pressure gradients and localized pressure concentrations. In stark contrast, an effective keel stabilizes the entire cross-sectional pressure distribution by creating persistent low-pressure zones at the cores of the stable vortices it induces [30], making the pressure profile smoother (Figure 11b,d).

**Figure 12:**
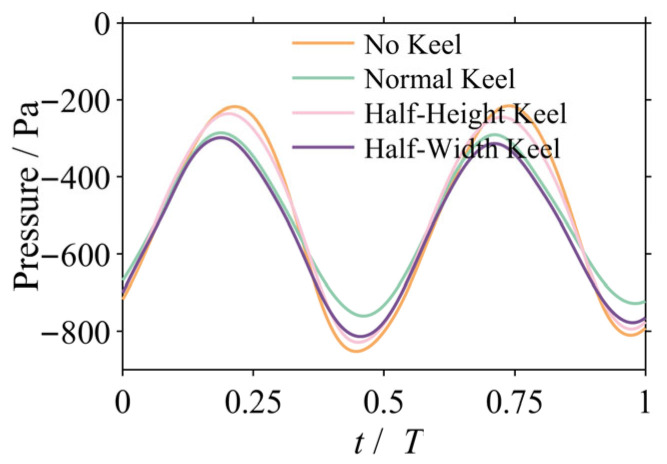
Comparison of area-averaged pressure fluctuation on the tail surface for the 2 Hz case.

To quantitatively validate this stabilizing effect, the area-averaged pressure fluctuation on the tail surface was monitored over one oscillation cycle for the 2 Hz case (Figure 12). As illustrated, the No Keel configuration (orange line) exhibits intense and irregular pressure pulsations. In contrast, the pressure fluctuation curve for the Normal Keel configuration (green line) is not only smaller in amplitude but also smoother in profile. Quantitative calculations reveal that the Normal Keel configuration reduces the standard deviation of the pressure fluctuation from 223.37 Pa to 162.51 Pa, which is a significant reduction of 27.2%. This result provides strong quantitative evidence that the keel stabilizes the flow field by substantially suppressing local pressure pulsations, thereby reducing the unsteady disturbance moments acting upon it.

#### 3.3.3. Analysis of Surface Pressure Distribution on the Robotic Dolphin

**Figure 13:**
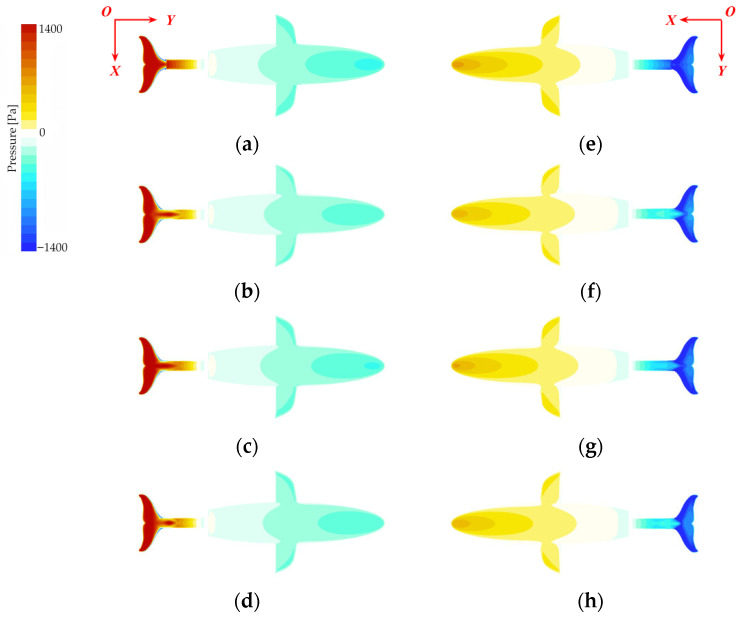
Surface pressure contours of the bionic dolphin robot: (**a**–**d**) pressure distribution on the upper surface for the No Keel, Normal Keel, Half-Height Keel, and Half-Width Keel models, respectively. (**e**–**h**) Corresponding pressure distribution on the lower surface.

The optimization of local flow and pressure fields ultimately manifests in the global pressure distribution over the entire fuselage. Figure 13 displays the surface pressure contours for the four models during the peak pitching moment for the 2 Hz condition, revealing a degree of optimization that perfectly aligns with the stability ranking.

The baseline No Keel model, shown in Figure 10a,e, exhibits intense pressure concentrations. At this stage, large areas of the caudal fin’s upper surface, the pressure side, experience high pressure, while the lower surface, the suction side, corresponds to strong negative pressure. This significant difference in pressure creates a strong pressure couple at the tail—the primary source of the pitching disturbance moment. Concurrently, this pressure disturbance propagates upstream, leading to a correspondingly non-uniform pressure distribution on the torso and head.

In contrast, models equipped with a keel exhibit markedly improved pressure distribution. For the most stable Normal Keel model, illustrated in Figure 10b,f, the high-pressure region on the upper caudal fin is significantly reduced and concentrated only at the fin’s root. The intensity of the negative pressure on the lower surface is also effectively suppressed, greatly attenuating the strength of the tail’s pressure couple. More importantly, this optimization is reflected upstream on the torso and head: the pressure on the upper surface becomes closer to neutral; the negative pressure distribution on the lower surface grows more uniform and moderate; and the pressure gradient in the head region is significantly reduced. The pressure distributions of the Half-Width and Half-Height models fall between these extremes, with the degree of optimization corresponding to their respective stability performances.

In summary, the caudal keel stabilizes the head through a complete physical chain of events: first, it achieves synergistic attenuation of the moment components at their physical source through flow field reconstruction and pressure field optimization; this quantitative attenuation ultimately leads to a reduction in the net pitching moment, thereby stabilizing the head attitude.

### 3.4. Analysis of Propulsive Energetics

**Figure 14:**
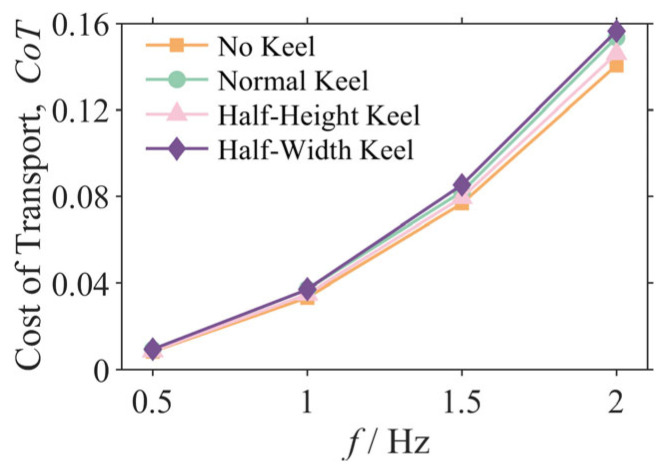
Cost of Transport (CoT) as a function of oscillation frequency.

To evaluate the energetic implications of the caudal keel, the Cost of Transport (CoT) for each configuration was calculated and compared with the results summarized in Figure 14. CoT is a dimensionless metric that quantifies the energy required to move a unit weight over a unit distance, where lower values indicate a higher energetic economy.

The analysis reveals that the addition of a caudal keel generally incurs an energetic penalty. For the representative 2 Hz condition, while the Normal Keel configuration delivered the best stability, its Cost of Transport (CoT = 0.153) was approximately 9.1% higher than that of the No Keel configuration (CoT = 0.141).

This result highlights a critical design trade-off between stability and energetic economy. The physical reason for this trade-off is that while the keel reorganizes the flow field to enhance stability, its increased wetted surface area also introduces additional frictional drag. To overcome the increased total drag, the robot must expend more propulsion power, leading to a higher CoT. This finding is crucial for the optimal design of future biomimetic underwater vehicles. For missions requiring high-precision sensing and attitude holding (e.g., underwater mapping and target identification), sacrificing some energetic economy for a critical gain in head stability is a worthwhile trade-off. Conversely, for long-endurance cruising missions, a configuration with lower energy consumption might be preferable.

### 3.5. Systematic Investigation of Keel Height

To elucidate the functional relationship between keel height, as the dominant geometric parameter, and stability, a systematic numerical investigation was conducted. While maintaining a constant keel width, the evolution of the standard deviation for the simulated head pitch (*σ_sim_*) was examined over a non-dimensional height (h/c) range from 0 (No Keel) to 0.20.

**Figure 15:**
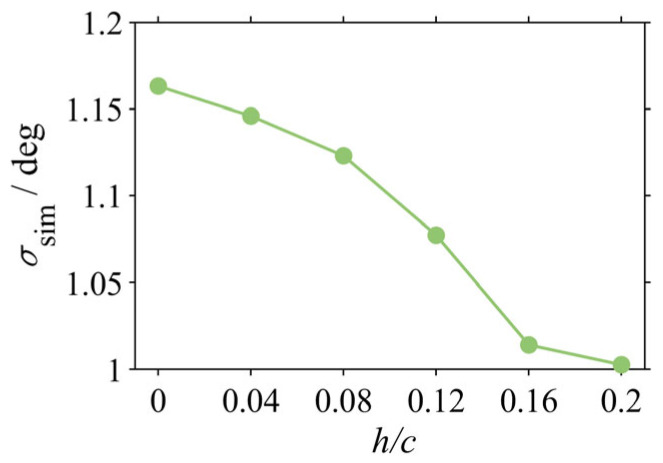
Effect of keel height on head stability.

The results, presented in Figure 15, reveal a monotonic but non-linear relationship where head stability improves with increasing keel height. As h/c increases from 0 to 0.16, *σ_sim_* is reduced by approximately 12.8% (from 1.16° to 1.01°), demonstrating that increasing keel height is an effective strategy for enhancing passive stability within this range.

Crucially, this investigation reveals a phenomenon of diminishing returns. The stability gained from increasing h/c from 0.16 to 0.20 is only an additional 1.1%, indicating that the stabilizing effect approaches an asymptotic saturation point. This finding has significant implications for engineering design: while keel height is a critical parameter for stabilization, an effective design range exists. Under the conditions of this study, further increases in height beyond an h/c of approximately 0.16 yield minimal performance benefits while incurring penalties in structural mass and frictional drag. A trade-off must therefore be considered to achieve an optimal balance between performance and cost.

## 4. Discussion

To properly interpret the findings and contributions of this study, it is essential to consider the inherent simplifications of our model and the specific hydrodynamic regime investigated. We must also highlight the novel aspects of this study compared to previous works. The investigations were conducted within a Reynolds number (Re) range (approx. 1.0 × 10^5^ to 3.3 × 10^5^) that confirmed turbulent flow, but which is lower than those of adult dolphins at high speeds (>10^7^) and more representative of juvenile dolphins or adults during slow cruising [31]. Similarly, the Strouhal number (St), ranging from 0.68 to 0.83, is notably higher than the 0.2–0.4 range associated with optimal propulsive efficiency [32]. This high-St regime, likely a consequence of the simplified kinematics, involves strong vortex shedding. While our findings robustly demonstrate the keel’s effectiveness in effecting these strong vortices, its performance characteristics within the biologically optimal, lower-St regime remain an important question for future research [33]. Furthermore, the simplified rigid-body, single-joint model chosen to isolate the keel’s fundamental effects neglects potential modulations from the continuous body flexibility inherent in biological counterparts. Finally, the quiescent water environment provided a necessary controlled baseline but excludes the complexities of real-world currents, waves, or turbulence, under which the strategy’s robustness needs verification. Verifying the robustness of the stabilization mechanism in more complex, disturbed flow fields is a critical area for subsequent investigations.

Despite these contextual factors, this study offers significant advancements. While prior research acknowledged the keel’s potential in flow optimization and stability, often focusing on propulsion or active control, our work provides the first quantitative evidence of a dual stabilization mechanism. We demonstrated through a combined experiment and CFD, particularly the frequency domain analysis (Section 3.2.2), that an effective keel synergistically attenuates the disturbance moment intensity (amplitude) and enhances its spectral purity by suppressing high-frequency harmonics and noise. This elucidation links systematic vortex reorganization to signal quality improvement, presenting a more nuanced understanding compared to previous amplitude-focused or qualitative descriptions.

Moreover, unlike earlier studies with limited geometric tests, our systematic investigation (Section 3.5) identified non-dimensional height (h/c) as the dominant parameter for stability. We revealed diminishing returns, establishing a concrete design guideline (an optimal range near h/c ≈ 0.16). This provides direct, quantitative criteria that are valuable for engineering optimization and were previously lacking in research. Crucially, by quantifying the energetic cost via the Cost of Transport (CoT) (Section 3.4), this study established the quantifiable trade-off between stability enhancement (approx. 20.9% gain) and energetic cost (approx. 9.1% increase). While the benefits of passive methods are known, this explicit quantification for a bio-inspired structure offers critical data for mission-specific AUV design—balancing the competing demands of high-precision sensing versus long-endurance cruising. In essence, by integrating experimental validation, detailed CFD, spectral analysis, parametric study, and energetic assessment, this work provides an extensive physical insight into and practical design principles for passive stabilization in biomimetic underwater vehicles.

## 5. Conclusions

This study presents a systematic investigation of the passive stabilization mechanism conferred by a caudal keel on a self-propelled biomimetic robot, employing a synergistic combination of experimental validation and numerical simulation. This research elucidates the keel’s governing physical principles and establishes key design criteria. The principal findings are summarized as follows:

(1) The caudal keel is an effective passive stabilization device, with its efficacy being pronounced at high frequencies. Experiments demonstrated that at 2 Hz, the Normal Keel configuration reduced the standard deviation of the head pitch angle by 20.9%, which is a highly significant result (*p* < 0.01).

(2) The principal stabilization mechanism is attributable to the dual-modal suppression of both the intensity and the spectral content of the disturbance moment signal. Frequency domain analysis (FFT) revealed that an effective keel attenuates the primary disturbance moment at the driving frequency (2 Hz) while concurrently suppressing spurious high-frequency harmonics and broadband stochastic noise through the systematic reorganization of the caudal vortex structures.

(3) Keel height was identified as the dominant geometric parameter governing stability, with its efficacy characterized by a non-linear relationship with diminishing returns. A systematic investigation demonstrated that while stability improves monotonically with non-dimensional height (h/c), the rate of gain diminishes, approaching an asymptotic saturation point beyond h/c ≈ 0.16. This finding provides a critical criterion for structural optimization, establishing an optimal design range wherein performance benefits are balanced against material and hydrodynamic costs.

(4) A quantifiable design trade-off between stability augmentation and energetic economy was established. Analysis of the Cost of Transport (CoT) revealed that the most stable Normal Keel configuration incurred an approximate 9.1% increase in energy expenditure at 2 Hz relative to the baseline No Keel case. This quantified relationship is integral to the mission-specific optimization of future AUVs, particularly in balancing the competing demands of high-precision sensing and long-endurance cruising.

In summary, the physical mechanisms and design principles elucidated in this study establish a theoretical framework for the development of highly stable biomimetic underwater vehicles. Subsequent investigations should focus on verifying the robustness of this passive stabilization strategy in more complex environmental settings, such as exposure to currents and waves, and exploring the potential of hybrid control schemes that can integrate this passive approach with active control systems.

## Figures and Tables

**Table 1:** Geometric parameters of the different caudal keel designs, non-dimensionalized by the chord length (c = 275 mm).

Model Name	Height (mm)	Width (mm)	h/c	w/c
No Keel	-	-	-	-
Normal Keel	44	50	0.160	0.182
Half-Height Keel	22	50	0.080	0.182
Half-Width Keel	44	25	0.160	0.091

**Table 2:** Grid independence study results for the 2 Hz No Keel case.

Grid Scheme	Number of Cells (Million)	Amplitude of *M_Y_* (N·m)	Difference from Previous Grid (%)
Coarse	1.19	83.21	-
Medium	2.20	83.23	0.024
Fine	4.49	83.19	−0.048

**Table 3:** Time-step independence study results based on the medium grid for the 2 Hz No Keel case.

Time-Step Scheme	Time Step Δ*t*	Amplitude of *M_Y_* (N·m)	Difference from Previous Grid (%)
Coarse	T/50	83.26	-
Medium	T/100	83.23	−0.036
Fine	T/200	83.25	0.024

## Data Availability

The data presented in this study are available on request from the corresponding author.
